# An evaluation of quality assurance guidelines comparing the American College of Radiology and American Association of Physicists in Medicine task group 284 for magnetic resonance simulation

**DOI:** 10.1002/acm2.13730

**Published:** 2022-07-18

**Authors:** Jacob S. Buatti, Kyle J. Gallagher, Isaac Bailey, Thomas Griglock, Malcolm Heard

**Affiliations:** ^1^ Department of Radiation Medicine Oregon Health and Science University Portland Oregon USA; ^2^ Department of Diagnostic Radiology Oregon Health and Science University Portland Oregon USA

**Keywords:** external beam radiation therapy, magnetic resonance imaging, magnetic resonance simulation, MR‐SIM, MR simulator, quality assurance, TG‐284

## Abstract

**Purpose:**

The purpose of this study was to evaluate similarities and differences in quality assurance (QA) guidelines for a conventional diagnostic magnetic resonance (MR) system and a MR simulator (MR‐SIM) system used for radiotherapy.

**Methods:**

In this study, we compared QA testing guidelines from the American College of Radiology (ACR) MR Quality Control (MR QC) Manual to the QA section of the American Association of Physicists in Medicine (AAPM) Task Group 284 report (TG‐284). Differences and similarities were identified in testing scope, frequency, and tolerances. QA testing results from an ACR accredited clinical diagnostic MR system following ACR MR QC instructions were then evaluated using TG‐284 tolerances.

**Results:**

Five tests from the ACR MR QC Manual were not included in TG‐284. Five new tests were identified for MR‐SIM systems in TG‐284 and pertained exclusively to the external laser positioning system of MR‐SIM systems. “Low‐contrast object detectability” (LCD), “table motion smoothness and accuracy,” “transmitter gain,” and “geometric accuracy” tests differed between the two QA guides. Tighter tolerances were required in TG‐284 for “table motion smoothness and accuracy” and “low contrast object detectability.” “Transmitter gain” tolerance was dependent on initial baseline measurements, and TG‐284 required that geometric accuracy be tested over a larger field of view than the ACR testing method. All tests from the ACR MR QC Manual for a conventional MR system passed ACR tolerances. The T2‐weighted image acquired with ACR sequences failed the 40‐spoke requirement from TG‐284, transmitter gain was at the 5% tolerance of TG‐284, and geometric accuracy could not be evaluated because of required equipment differences. Table motion passed both TG‐284 and ACR required tolerances.

**Conclusion:**

Our study evaluated QA guidelines for an MR‐SIM and demonstrated the additional QA requirements of a clinical diagnostic MR system to be used as an MR‐SIM in radiotherapy as recommended by TG‐284.

## 1 INTRODUCTION

Regular quality assurance (QA) testing of magnetic resonance (MR) imaging systems ensures that the system is operating safely and produces images that maintain a satisfactory level of performance acceptable for diagnosing diseases or planning treatment interventions. A robust and comprehensive QA program should encompass tests that monitor image quality, mechanical components, safety equipment, safety systems, axillary equipment, and other MR system characteristics. QA guides have been created to aid hospital staff, including medical physicists and MR technicians, in creating and executing QA programs for MR systems. However, these guides have not traditionally been designed for testing radiation therapy‐specific image quality metrics and have not included new modalities like the MR simulator (MR‐SIM).

The American College of Radiology (ACR) establishes and regularly updates QA testing instructions and performance criteria for commercial MR modalities used for patient imaging. The most recent released document, titled “2015 magnetic resonance imaging (MRI) Quality Control (QC) Manual,” defines the expectations of an MR program and, of concern for this study; the minimum level of performance the scanner must meet to appropriately and safely image patients. However, performance characteristics from the ACR MR QC manual are specific to diagnostic radiology purposes and not for radiotherapy applications. The ACR MR QC guide states, “the qualified medical physicist/MRI scientist may determine that a more strict action limit should be put in place,” for example, stereotactic radiation therapy planning or for co‐registering images from different scanners and time frames^1^.

In modern radiotherapy, MR images are acquired for developing treatment plans unique to the patient's disease type, location, size, and proximity to radiosensitive organs. MR images provide superior soft tissue contrast compared to computed tomography (CT) that benefits the physician's ability to accurately delineate the tumor volume and surrounding normal tissues^2^, ^3^. These contours are then used to prescribe and optimize where radiation will be delivered to the patient. For some disease sites, previous studies have demonstrated that using MR images for contouring and treatment planning may reduce treatment‐related toxicities by improving the localization of the gross tumor volume (GTV) and organs at risk^2^, ^3^. Additionally, the superior soft‐tissue characteristics from MR images may be used to escalate dose in certain regions of the GTV which may improve clinical outcomes^2^, ^4^.

The conventional workflow for using MR images in radiotherapy treatment planning has been to avoid directly using MR imaging for treatment planning and instead has relied on CT images. In this conventional CT‐based workflow, CT images of the patient have been defined as the “reference geometry” due to the high geometric fidelity of the CT images and electron density information needed for accurate dosimetric calculations in heterogeneous tissues. Replicative treatment positioning using immobilization devices, a flat tabletop, and external laser positioning systems (ELPSs) have all been achieved in CT‐simulation (CT‐SIM). CT‐SIM systems are equipped with identical positioning equipment to the treatment room to ensure that the images used for treatment planning are representative of the patient's position during treatment. An increase in geometric uncertainties occurs when registering MR images to CT images due to differences in patient positioning, no use of immobilization devices, and nonspecific states of interest (e.g., breath holds) in MR images.

Improvements to MR systems including equipment hardware and image processing algorithms have led to a reduction in geometric uncertainties and the ability to synthesize CT data from MR image data^1^. For treatment planning, increased accuracy for MR imaging can be performed using an MR simulator (MR‐SIM), equipped with simulation accessories including MR compatible immobilization devices, immobilization device compatible RF coils, ELPS, flat tabletop, and a wide bore. Already several commercial synthetic CT products are available with limited approval by the Federal Drug Administration for use in MR‐only treatment planning^2^, ^5^, ^6^. As improvements continue, the use of MR‐SIM systems and MR‐only treatment planning is expected to increase^7^.

Presently, dedicated MR modalities in radiation oncology departments are not common and MR‐SIM systems, in general, are uncommon. Typically, MR systems and resources are shared with diagnostic imaging departments and are tested by diagnostic physicists. Aside from end‐to‐end testing for specific treatment techniques (e.g., stereotactic radiation surgery), MR performance is often not evaluated by therapy physicists, who traditionally do not have expertise in MRI QC. Therefore, a multidisciplinary team to support the MR simulator may be required.

To ensure that MR images acquired with an MR‐SIM modality are meeting the image quality and safety requirements for radiotherapy treatment planning, a robust QA program should be implemented. For this purpose, the American Association of Physicists in Medicine (AAPM) Task Group 284 (TG‐284) released a report detailing recommendations for QA of an MR‐SIM system, including respective frequencies and tolerances for each test. The suggested QA program of TG‐284 overlaps with the QA required for ACR MR accreditation. However, similarities and differences between the two QA programs have yet to be examined.

The purpose of this study was to evaluate the QA guidelines for MR systems used for both diagnostic imaging and MR simulation. Specifically, we compared the QA testing requirements, procedures, frequency, and tolerances between TG‐284 and the ACR MR QC Manual. Further ACR testing was performed on a clinical diagnostic MR system and the results were compared to recommendations from TG‐284. For institutions with both radiation oncology and diagnostic imaging physics expertise, a study of this nature may be beneficial for communication between departments and for establishing an appropriate QA program for MR‐SIM systems or general MR use in radiotherapy.

## 2 MATERIALS AND METHODS

### 2.1 QA test comparison between ACR and TG‐284

QA guidelines for MR systems in diagnostic and radiotherapy applications were analyzed to identify similarities and differences in overall testing scope, and performance characteristics required of the two modalities. For an MR‐SIM system used in radiotherapy, the QA section of TG‐284 was selected for analysis because of its specificity to MR‐SIM systems, image quality emphasis for radiation oncology treatment planning, the inclusion of QA for MR‐SIM specific equipment, and recent publication. For a conventional MR system in diagnostic radiology, the ACR MR QC Manual was chosen for analysis. The ACR MR QC Manual is used at our institution and many other institutions to ensure the reliable and appropriate performance of conventional MR systems. ACR MR Accreditation is widely accepted as the standard for MR system performance. Further, TG‐284 refers to the ACR QC Manual for more detailed information regarding relevant tests (e.g., image constancy). Therefore, these two relevant QA guides were chosen for evaluation in this study.

A comparison of the two QA guides was performed in a multistep process. First, a complete list of all tests described in each report was made. Each list consisted of individual QA tests, the recommended testing frequency for each test, and their respective action tolerances. Next, the QA testing lists from each guide were compared to identify existing similarities and differences. Three categories for testing comparison were created. First, “identical tests” were tests that evaluated the same performance trait (e.g., low‐contrast object detectability [LCD]) and were included in both QA guides. Second, “excluded tests” were tests that were included in the ACR MR QC Manual but not in TG‐284. Third, “new tests” were tests that were described in TG‐284 but not in the ACR MR QC Manual. Lastly, identical tests were compared further by testing frequency and action limits.

### 2.2 Performance of a clinical diagnostic MR scanner

To examine the performance of a clinical MR scanner, results from an annual MR QA were evaluated using TG‐284 QA action limits. The annual QA for a commercial 3.0 T, 70 cm diameter bore MR system (Ingenia, Philips Medical Systems, Cleveland, OH) was performed by an ABR‐certified imaging physicist following ACR MR QC procedures and with the Large ACR MR Phantom (American College of Radiology, Reston, VA). For the requirements of ACR MR testing, T1‐ and T2‐weighted pulse sequences defined by the ACR, as well as T1‐ and T2‐weighted sequences commonly used at our institution, were acquired and evaluated for image quality and other tests (Table 1).

**TABLE 1:** Description of MR imaging studies for American College of Radiology (ACR) annual quality assurance (QA)

**Study**	**Pulse sequence**	**TR** **^a^** **(ms)**	**TE^b^ (ms)**	**FOV^c^ (cm)**	**#Of slices**	**Slice thick‐ness (mm)**	**Slice gap (mm)**	**NEX^d^**	**Matrix (pixels)**	**BW^e^(kHz)**	**Scan time (min:sec)**
ACR Sagittal Localizer	90° Spin Echo	200	20	25	1	20	NA	1	256×256	55.8	0:53
ACR Axial T1	90° Spin Echo	500	20	25	11	5	5	1	256×256	55.8	2:10
ACR Axial T2	90° Spin Echo	2000	20 80	25	11	5	5	1	256×256	55.8	8:32
Axial T1 Brain Scan	90° Spin Echo	500	10	23	11	5	5	1	256×190	49.8	0:59
Axial T2 Brain Scan	90°Fast Spin Echo	3000	80	23	11	5	5	1	420×288	113.7	0:39

^a^ Repetition time (TR), ^b^Echo time (TE), ^c^Field of view (FOV) (cm), ^d^Number of excitations (NEX), ^e^Bandwidth (BW) (kHz/pixel).

Three QA tests with different acceptable performance criteria were considered and evaluated using both ACR and TG‐284 tolerances. First, low contrast object detectability (LCD) was evaluated using both ACR T1 and T2 images (Table 1) and the testing method for annual ACR QA. The number of complete discernible LCD spokes in the LCD region of the ACR phantom was counted and summed to determine the LCD resolution of the system. Second, transmitter gain measurements were recorded from the MR system console after imaging the Large ACR Phantom with a head coil.

Table motion smoothness and accuracy were measured using two different methods. For ACR accreditation, table motion accuracy was measured following ACR MR QC procedures. The Large ACR phantom was aligned with the external isocenter laser of the MR system and translated to the isocenter of the MR. Localizer scans in sagittal, coronal, and axial planes were acquired. The distance between the isocenter of the phantom and the isocenter of the image was measured using a ruler tool in the Philips MR imaging software to determine the table motion offset and accuracy. For TG‐284 table motion and accuracy testing, a direct measurement was acquired. A marker, placed at a zeroed position, was moved into and out of the bore of the magnet at 150 mm and 300 mm fixed intervals and measured with a ruler to determine the motion accuracy of the table.

## 3 RESULTS

### 3.1 QA test comparison between ACR and TG‐284

QA testing scope and testing frequency differences between the ACR MR QC Manual and TG‐284 are outlined in Table 2A,B for MR Technologists and Table 2C for physicists. Five tests from the ACR MR QC Manual were excluded from TG‐284 (excluding individual items of the visual checklist). TG‐284 did not include slice‐thickness accuracy, slice position accuracy, soft‐copy/monitor control, an annual review of the visual checklist, and an annual review of the MR safety program (Table 2C). Additionally, 10 items from the ACR MR QC visual checklist were excluded from the scope of TG‐284 QA testing (Table 2B).

**TABLE 2:** A Quality assurance testing frequency recommendations for MR technologists

		Frequency	
	Quality assurance (QA) tests for MR technologists	TG‐284	ACR
1	Functionality of patient communication and monitoring	D	W^v^
2	Emergency cart or emergency couch release	D	W^v^
3	Safety signage	D	W^v^
4	Check bore for presence of foreign metal objects	D	W^v^
5	External laser agreement with imaging plane	D	^*^
6	Transmit gain central frequency	D	W
7	Basic coil signal‐to‐no SNR check	D	W
8	Basic spatial fidelity check	D	W
9	High‐contrast spatial resolution	^*P^	W
10	Low‐contrast detectability	^*P^	W
11	Visual checklist	^*^	W
12	Artifact evaluation	^*P^	W

B American College of Radiology (ACR) visual checklist items and testing frequency recommendations for MR technologists			
		Frequency	
	ACR visual checklist items	TG‐284	ACR
1	Functionality of patient communication and monitoring	D	W
2	Emergency cart or emergency couch release	D	W
3	Safety signage	D	W
4	Check bore for presence of foreign metal objects	D	W
5	Table position and other displays	^**^	W
6	Alignment lights	^**^	W
7	Horizontal table motion smoothness and stability	^**^	W
8	Vertical table motion smoothness and stability	^**^	W
9	Laser camera	^**^	W
10	Light boxes	^**^	W
11	RF door contact	^**^	W
12	RF window‐screen integrity	^**^	W
13	Operator console switches/lights/meters	^**^	W
14	Room temperature/humidity	^**P^	W
15	Cryogen indicator	^**P^	W
16	Door indicator switch	^**^	W

C Quality assurance testing frequency recommendations for medical physicists or MR scientists			
		Frequency	
	QA tests for physicists or MR scientists	TG‐284	ACR
	**Image quality**		
1	Geometric accuracy	M	A
2	High contrast spatial resolution	M	A
3	Low contrast detectability	M	A
4	Artifact evaluation	M	A
5	Percent image uniformity (PIU)	M	A
6	Percent signal ghosting	M	A
7	Central frequency	M	A
8	Transmitter gain	M	A
	**Mechanical tests**		
9	Table movement smoothness and accuracy	M	A
10	Laser alignment with imaging isocenter	M	^***^
11	Laser movement smoothness and accuracy	M	^***^
	**Patient marking**		
12	Laser marking accuracy	M	^***^
	**System**		
13	Room temperature and humidity	M	V
14	Cold head operation	M	V
15	Cryogen level indicator	M	V
	**Other**		
16	Flexible radiofrequency coil testing	M	A
17	Transmitter and gain calibration	A	A
18	Magnetic field homogeneity (B_0_)	A	A
19	Rigid radiofrequency coil evaluation	A	A
20	Determine or verify external laser offset from MR isocenter	A	^***^
21	Soft‐copy (monitor) control	^***^	A
22	MR safety program assessment	^***^	A
23	Visual checklist review	^***^	A
24	Slice‐thickness accuracy	^***^	A
25	Slice‐position accuracy	^***^	A

^*^Test not identified in report; D, daily; W^v^, weekly test included in ACR visual checklist (Table 2B)^2^; W, weekly. ^P^Test performed by physicists for TG‐284 monthly QA (Table 2C).

^**^Test not identified in report; D, daily; W, weekly. ^P^Tests performed by physicists for TG‐284 monthly QA (Table 2C). Note: TG‐284 contains a visual checklist adapted from the ACR in the appendix of the report but does not identify the checklist as required component of QA or with a stated frequency.

^***^Test not identified in report; M, monthly; V, visual checklist ‐ tested weekly (Table 2B); A, annually.

Five new tests were described in TG‐284 and were specific to ELPS QA. “External laser agreement with imaging plane,” “laser alignment with imaging isocenter,” “laser movement and smoothness accuracy,” “determine or verify external laser offset from MR isocenter,” and “laser marking accuracy” were all added to QA for MR‐SIM specific equipment.

For all 23 identical tests identified, two had different action limits, one required different testing equipment, and two were possibly different depending on the tolerances defined by the vendor during commissioning. The two tests with different acceptable tolerances were “LCD” and “table motion smoothness and accuracy” (Table 3A,B). TG‐284 geometric accuracy testing required that the FOV be greater than or equal to 25 cm, while the ACR only required that the test be performed over the smaller dimensions of the phantom (Large ACR Phantom diameter = 19 cm, length = 14.8 cm).

**TABLE 3:** A Image quality quality assurance (QA) testing tolerances from American College of Radiology (ACR) and TG‐284 using the ACR Large MRI Phantom

		Tolerances
QA tests	TG‐284	ACR
**Image quality**		
Geometric accuracy	±2 mm across 25‐cm field of view (FOV) with phantom >30 cm in diameter	±2 mm using Large ACR Phantom
High contrast spatial resolution	≤1.0 mm	≤1.0 mm
Low contrast detectability	21 (0.3 T) to 36 (1.5 T) total spokes, 40 spokes (3 T)	≥7 spokes, 9 spokes preferred (1.5 T), ≥37 spokes (3 T)^b^
Artifact evaluation	No observable artifacts	No observable artifacts
Percent image uniformity (head coil)	≥87.5% for 1.5 T, ≥82.0% for 3.0 T	≥87.5 % for 1.5 T, ≥82.0 % for 3.0 T
Percent signal ghosting	≤2.5%	≤2.5 %
Central frequency	Vendor‐specified	Vendor‐specified (i.e. ± 500 Hz ^a^)
Transmitter gain	±5% from baseline	Vendor‐specified (i.e. ± 0.05 dB ^a^)
Flexible RF coil testing	Individual elements, exceeds minimum vendor‐specified threshold	Vendor‐specified

B Mechanical, patient marking, system, and other quality assurance (QA) testing tolerances from American College of Radiology (ACR) and TG‐284		
	Tolerances	
QA tests	TG‐284	ACR
**Mechanical tests**		
Table movement smoothness and accuracy	±1.0 mm from set distances	±5.0 mm from set distance
Laser alignment with imaging isocenter	±2.0 mm from expected distance offsets	NA
Laser movement smoothness and accuracy	±2.0 mm from set distances	NA
**Patient marking**		
Laser marking accuracy	±2.0 mm	NA
**System**		
Room temperature and humidity	Functional	Functional
Cold head operation	Functional	Functional
Cryogen level indicator	Functional	Functional
**Other**		
Magnetic field homogeneity	Vendor specified across a specified DSV or <0.5 ppm volume root mean square (VRMS) over a 35 cm DSV	Vendor specified across a specified DSV (e.g. <2 ppm VRMS over a 24‐cm DSV^c^)
RF coil evaluation	Coil and vendor dependent, ± one standard deviation from baseline for SNR, percent image uniformity, and percent signal ghosting	Coil and vendor specified for SNR, percent image uniformity, and percent signal ghosting
External Laser Alignment with Isocenter	≤1 mm	NA

^a^ Tolerance for Philips Ingenia 3.0 T system used for testing at our institution.

^b^ Tolerances are specified over all slices and for all 40 spokes per the ACR's measurement method for annual testing^2^.

^c^ Vendor‐specified tolerance for Philips Ingenia 3.0 T system used for testing at our institution.

Abbreviations: DSV, diameter spherical volume; NA, not applicable.

Transmitter gain action limits may be stricter for MR‐SIM systems than for conventional MR systems. TG‐284 recommended that transmitter gain be within 5% of the baseline measurement taken during commissioning, while the ACR did not specify an action limit for conventional MR systems. Transmitter gain tolerances may be set by the system vendor and can vary from machine‐to‐machine. For a clinical 3 T system at our institution, transmitter gain was required to be within 0.05 dB. This equated to a 6.06% deviation from the baseline. Depending on the system and baseline evaluation of transmitter gain, a 5% tolerance may be more rigorous.

Magnetic field homogeneity action limits could not be directly compared between the two QA guides. TG‐284 recommended that magnetic field homogeneity tolerance be set by the system manufacturer or be within 0.5 ppm volume root mean square (VRMS) over a 35‐cm diameter spherical volume (DSV). Similarly, the ACR stated that the tolerance be set by the manufacturer, but no alternative tolerance was stated. For the clinical MR system tested at our institution, the action limit defined during commissioning was determined to be 2 ppm VRMS over a 24‐cm DSV. This was determined using the bandwidth difference method described by Chen et al. in axial, sagittal, and coronal orientations and using a Siemens 24 cm DSV spherical phantom filled with distilled water and doped with NiSO_4_ ^9^. For wide bore systems used for treatment planning, a larger DSV may be required.

Geometric accuracy could not be evaluated due to equipment specification differences. For ACR testing, the length and diameter of the ACR phantom are measured and compared to known values (Large ACR Phantom diameter = 19 cm, length = 14.8 cm). For TG‐284, a phantom greater than 30 cm in diameter and width was recommended for measuring geometric accuracy across an FOV greater than or equal to 25 cm (Table 3A). While the tolerances for both ACR and TG‐284 geometric accuracy are both 2 mm, the FOV and equipment required for the measurement is different.

### 3.2 Performance of a clinical diagnostic MR scanner

The performance of a clinical diagnostic MR scanner at our institution was investigated by evaluating the ACR‐based annual MR QA using TG‐284 action limits. This evaluation was specifically done for the three tests that were previously identified as having stricter, or potentially stricter, action limits in TG‐284. These tests included LCD, table motion smoothness and accuracy, and transmitter gain.

For LCD test performed using the clinical MR scanner at our institution, 40 LCD spokes and 38 LCD spokes were observed in the ACR T1‐weighted and T2‐weighted images, respectively (Table 4). The number of observable spokes for both image types was within the 37 spoke requirement for 3 T magnets per ACR. For TG‐284 recommendations, the T1‐weighted image but not the T2‐weighted image met the 40 observable recommendations.

**TABLE 4:** Low‐contrast detectability measurements and evaluation with American College of Radiology (ACR) and TG‐284 quality assurance (QA) tolerances

Image series	Slice number	Total number of spokes	ACR^a^	TG‐284^b^
ACR T1	8‐11	40	PASS	PASS
ACR T2	8‐11	38	PASS	FAIL

^a^ ACR passing criteria for LCD were 37 or more total spokes.^2^

^b^ TG‐284 LCD passing criteria for a 3.0 T scanner were 40 total spokes.^2^

For transmitter gain, a measurement of 0.8668 dB was recorded from the MR console. The baseline gain measurement for the scanner was 0.8252 dB. Transmitter gain was within the 0.05 dB tolerance set during commissioning for ACR reporting. The percent difference between the measurement and baseline was 5.0%, which is at the 5% tolerance of TG‐284 (Table 5) and would be considered passing for clinical purposes.

**TABLE 5:** Transmitter gain (TG) measurement and evaluation with American College of Radiology (ACR) and TG‐284 quality assurance (QA) tolerances

Baseline TG Measurement (dB)	Annual TG Measurement (dB)	Relative Difference (%)	Absolute Difference (dB)	ACR^a^	TG‐284^b^
0.8252	0.8668	5.0 %	0.0416	PASS	PASS

^a^ ACR transmitter gain tolerance was ±5.0 dB from the baseline.^2^

^b^ TG‐284 transmitter gain tolerance was ±5 % from the baseline.^3^

Table motion accuracy was assessed by translating the table into and out of the bore with programmed distances of 150 mm and 300 mm and comparing these programmed translations against measured values with a ruler. Discrepancies in measured and programmed table translations were less than 1 mm and, therefore, were within the 1‐mm action limit of TG‐284 and the 5‐mm limit of the ACR requirements.

## 4 DISCUSSION

### 4.1 QA test comparison between ACR and TG‐284

The comparison of the two QA guides outlines the similarities and differences between the QA testing regimens. This comparison displays how the two QA guides for an MR‐SIM and conventional MR system vary regarding QA tests, testing frequencies, and acceptable tolerances for characterizing system performance.

Only three QA tests that were included in both reports had the same rate of testing frequency. The remaining tests required more frequent testing for an MR‐SIM system. In general, weekly and annual tests from the ACR MR QC Manual were increased to daily and monthly, respectively, for identical tests. This proposed testing schedule is reminiscent of QA testing for a CT‐SIM system and may serve to ensure that system performance is meeting requirements for radiotherapy treatment planning on a more regular basis in comparison to only once per year. A more frequent QA plan increases the likelihood of observing system deficiencies and ensures that images maintain an appropriate level of quality—which is imperative for treatment planning purposes.

Although increased QA testing frequencies have been recommended by TG‐284, more frequent testing will undoubtedly increase the workload of the medical physicists. TG‐284 estimates that monthly QA testing takes about 1 h, while daily QA testing is less than 30 min. This estimation may account for using weekly ACR MRI QA results to evaluate central frequency, transmitter gain, flexible RF coil testing, and geometric accuracy, which saves time. Regardless, increased testing time combined with the typical heavy workloads of clinical MR systems, will possibly create a logistical challenge to complete the more frequent QA required of the MR‐SIM. Institutions utilizing a diagnostic MR scanner for simulation purposes will be required to account for the increased QA requirements in the MR system schedule.

The visual checklist items from the ACR MR QC Manual were not explicitly addressed in TG‐284. The ACR requires that the 16 individual tasks be completed weekly and is included as a singular test in the ACR MR QC Manual (Table 2A). TG‐284, however, does not definitively recommend these items as one of the specifically outlined daily, monthly, or annual QA tasks. Instead, TG‐284 incorporates a visual checklist in the appendix of the report that is meant to serve as an example template for monthly tests. Given the importance of the visual checklist items as it relates to safe operation of the system, the omitted items in Table 2B should be performed by MR technologists as part of the weekly or daily MR‐SIM QA per ACR guidelines.

Another major difference between the QA guides for MR systems is the existence of pulse sequence requirements for QA testing. In contrast to the ACR MR QC Manual, TG‐284 does not require use of specific sequences. Rather, TG‐284 suggests a general sequence type (e.g., Gradient Echo or Spin Echo), for certain tests, such as magnetic field homogeneity. T1 and T2 sequences and the resulting image quality can vary widely depending on the image requirements of the study. For ACR MR testing, the required image sequences for evaluating performance are not representative of the scanner's best images and would not typically be used for patient imaging. Instead, these sequences are likely used to evaluate a baseline level of performance across institutions and scanner types. The lack of sequence requirements is a limitation in TG‐284 and becomes problematic for the evaluation of image quality QA tests (Discussion 4.2).

For MR‐SIM testing, more robust and radiotherapy‐specific sequences may be better suited for MR‐SIM QA compared to the ACR T1 and T2 sequences evaluated in this study (Table 1). Advanced sequences for QA testing are expected to improve image quality and, therefore, the testing results. If the goal of radiotherapy‐specific QA is to assess image quality and performance for treatment planning, evaluating image sequences used for treatment planning may be more appropriate. The physicist will need to decide whether ACR sequences should be used or if the site's radiotherapy specific protocols are better suited for MR‐SIM QA.

Lastly, extended use of flexible RF coils in MR SIM imaging exams will require more frequent QA testing and increase the QA efforts required for the system. For MR‐SIM imaging, flexible coils are used conform the coil to the patient in treatment position and to immobilization devices. The flexible nature of these coils makes them superior to rigid coils with respect to accommodating radiotherapy devices and positioning requirements. However, flexible coils are more prone to physical damage and may result in an increase incidence of image artifacts, decrease in signal‐to‐noise ratio (SNR), and degradation of image uniformity. While flexible coils are less common in diagnostic MR exams and require only annual testing for ACR certification, the increased use of flexible RF coils in MR simulation results in increased wear demanding more frequent testing of the devices.

The unique equipment requirements for an MR‐SIM system, like the large bore, make it a valuable tool for specialized imaging exams (e.g., bariatric MR imaging, imaging requiring unique positioning, or patients with claustrophobia). MR‐SIM systems may be utilized by multiple departments to maximize the workload and profitability of the system. As such, ACR MR accreditation of the MR‐SIM system would be required per typical billing requirements^9^. ACR MR QA certification of an MR‐SIM system would, therefore, require integrating the ACR and TG‐284 QA tests. This would involve completing the ACR tests at a minimum to maintain accreditation (e.g., including the use of ACR T1 and T2 sequences) and enhancing the QA program with the more frequent and extensive tests recommended by TG‐284 for MR‐SIM. Therefore, both the five new tests and the five excluded tests in TG‐284 would need to be performed to satisfy system‐specific QA requirements.

### 4.2 Performance of a clinical diagnostic MR scanner

ACR‐based annual QA testing results from a clinical diagnostic MR system at our institution were evaluated using performance standards from TG‐284 and the ACR MR QC Manual. Three tests were identified as having different performance tolerances. TG‐284 recommended stricter tolerances for LCD and table motion, whereas the tolerance for transmitter gain is possibility to be stricter depending on baseline measurements.

LCD QA failed the 40 total spoke tolerance of TG‐284 using the ACR T2 weighted sequence. While the ACR states that the user should proceed by evaluating LCD with institution‐defined T1 and T2 sequences upon failure, TG‐284 does not provide directions regarding how many sequence types should be evaluated, which sequences should be used for the test, or what actions should be taken if the test fails. The failure of the LCD QA demonstrates the ambiguity of the testing guidelines for image quality in TG‐284, whereas the ACR provides more specific requirements. If institution‐defined sequences should be used instead of ACR sequences for evaluating image quality tests such as LCD, the testing result can be expected to improve.

Since the publication of the ACR MR QC manual in 2015, updated versions of testing guidance documents have been released by the ACR, which have updated tolerances that may be made official as soon as Fall 2022. This includes the unofficial document, “Large and Medium Phantom Test Guidance for the ACR MRI Accreditation Program,” with updated LCD tolerances for MR systems with field strength between 1.5 T and 3 T. When made official, the new LCD tolerance for 1.5 T to 3.0 T magnets will be greater than or equal to 30 spokes for T1 sequences and greater than or equal to 25 spokes for T2 sequences. TG‐284 recommends a total spoke count of 36 spokes for 1.5 T magnets and for unspecified image sequence types (Table 3A). Regardless of the change, the requirements for MR‐SIM systems will remain stricter than that required by the ACR.

The table motion smoothness and accuracy QA test had a stricter tolerance for MR‐SIM systems and required different measurement methods. For ACR testing, table motion accuracy is not measured directly, and instead, an offset is calculated between the imaging isocenter and the center of the imaged ACR phantom. This procedure introduces larger uncertainties than a direct measurement of table motion. Conversely, TG‐284 recommends directly measuring table translation with a ruler and using fixed distance. Table translation during an MR imaging exam is not performed during image acquisition like in CT imaging but is used for translation of the patient between sequences. A direct measurement of the table translation accuracy using a ruler demonstrated that table motion on this diagnostic MR system was acceptable.

Transmitter gain performance tolerances for a conventional MR system and MR‐SIM are reported using two different scales in their respective QA guides (Table 3A). For ACR testing of our institution's clinical MR system (i.e., the Philips Ingenia 3.0 T scanner), a 0.05 dB tolerance was specified during commissioning, whereas a 5% tolerance was recommended by TG‐284 for all MR systems. Variations in transmitter gain and the resulting flip angles manifest as differences in image contrast. A 5% difference, which was found to be a stricter tolerance than 0.05 dB for the tested system, further ensures that appropriate image quality is maintained for treatment planning.

Geometric accuracy could not be evaluated using the TG‐284 tolerance because of equipment differences. Specifically, TG‐284 geometric accuracy tolerance is specified over a phantom with a diameter greater than 30 cm and across a 25‐cm FOV (Table 3A). These dimensions are greater than the dimensions of the Large ACR Phantom which, therefore, cannot be used for this test. Geometric accuracy across the entire patient is critical for strategically planning radiation doses in radiotherapy. A larger testing FOV ensures the spatial fidelity of the image at extended fields is appropriate. Because the Large ACR Phantom cannot be used for this test, institutions will be required to purchase a phantom that fits the needs for MR‐SIM QA.

Both ACR and TG‐284 QA tests of magnetic field homogeneity recommend that the tolerance be set by the vendor during commissioning (Table 3B). Following vendor recommended tolerances is widely accepted among institutions and accurately reflects a system's performance. However, TG‐284 suggests a second tolerance for MR‐SIM systems if the institution chooses not to use vendor recommendations. TG‐284 recommends that magnetic field homogeneity be less than 0.5 ppm VRMS over a 35‐cm DSV. The recommended larger DSV measurement for MR‐SIM systems is likely present due to the wide bore requirement of the system and the necessity for high spatial fidelity across the entire image field of view used for treatment planning. Furthermore, the magnetic field homogeneity typically deteriorates with increasing distance from the imaging isocenter^10^. For all MR systems evaluated at our institution, magnetic field homogeneity is tested across a 24‐cm DSV. For MR‐SIM applications and treatment planning for which fidelity of the body habitus is important for dose calculation, it would be beneficial for the tolerance to be specified over a larger DSV (e.g., 35 cm) to characterize the magnetic field across a greater FOV.

A key feature of this study is the evaluation of radiotherapy‐specific QA compared to the standard ACR QA required for diagnostic MR systems. For radiation medicine departments that rely on MR systems in diagnostic radiology departments, this study highlights some of the additional radiotherapy‐specific tests and imaging protocols recommended by TG‐284 compared to the ACR QA. If not directly involved in the ACR QA, the radiation therapy physicist should review the ACR results and perform additional radiotherapy‐specific tests to ensure appropriate MR performance. Moreover, a comprehensive QA process should be developed and implemented for diagnostic MR scanners used for therapy planning and should include the key QA tests and features recommended in TG‐284.

## 5 CONCLUSION

In this study, we evaluated the similarities and differences in QA guidelines for a conventional diagnostic MR system and an MR simulator used in radiotherapy, for which a robust comparison was done between the ACR MR QC Manual and the QA section of TG‐284. Specifically, the assessment showed that five tests from the ACR MR QC guide were excluded in TG‐284, five new tests were described in TG‐284 for MR‐SIM‐specific equipment, three tests that existed in both reports had different tolerances or measurement methods, and in general, there were considerable differences in testing frequency. Furthermore, annual MR QC testing results from a clinical diagnostic MR scanner at our institution were evaluated using the differing tolerances from the two QA guides. All QA tests met the minimum level of performance for ACR MR Accreditation while LCD QA and geometric accuracy tests failed TG‐284 tolerances and required different equipment, respectively. TG‐284 lacked imaging sequence guidelines for QA testing and failed to address whether T1‐weighted, T2‐weighted, or both sequences were required for image quality assessment. For MR‐SIM systems that are a shared resource between diagnostic radiology and radiation medicine, a comprehensive QA program and multidisciplinary approach is necessary as highlighted in this study.

## AUTHOR CONTRIBUTIONS

J. B., M. H., K. G., and T. G. conceived of and designed the presented project. J. B. and I. B. performed experiments. J. B. reviewed reports and wrote the manuscript. K. G., T. G., and M. H. supervised J. B. and provided feedback that shaped the research. K. G. and M. H. directed J. B. with radiotherapy expertise for the discussion. T. G. and I. B. provided imaging physics expertise to J. B. for the discussion.

## CONFLICT OF INTEREST

The authors have no relevant conflict of interest to disclose.
